# Binary dwarf mongoose optimizer for solving high-dimensional feature selection problems

**DOI:** 10.1371/journal.pone.0274850

**Published:** 2022-10-06

**Authors:** Olatunji A. Akinola, Jeffrey O. Agushaka, Absalom E. Ezugwu

**Affiliations:** 1 School of Mathematics, Statistics, and Computer Science, University of KwaZulu-Natal, Pietermaritzburg, KwaZulu-Natal, South Africa; 2 Department of Computer Science, Federal University of Lafia, Lafia, Nasarawa State, Nigeria; The University of Stirling, INDIA

## Abstract

Selecting appropriate feature subsets is a vital task in machine learning. Its main goal is to remove noisy, irrelevant, and redundant feature subsets that could negatively impact the learning model’s accuracy and improve classification performance without information loss. Therefore, more advanced optimization methods have been employed to locate the optimal subset of features. This paper presents a binary version of the dwarf mongoose optimization called the BDMO algorithm to solve the high-dimensional feature selection problem. The effectiveness of this approach was validated using 18 high-dimensional datasets from the Arizona State University feature selection repository and compared the efficacy of the BDMO with other well-known feature selection techniques in the literature. The results show that the BDMO outperforms other methods producing the least average fitness value in 14 out of 18 datasets which means that it achieved 77.77% on the overall best fitness values. The result also shows BDMO demonstrating stability by returning the least standard deviation (SD) value in 13 of 18 datasets (72.22%). Furthermore, the study achieved higher validation accuracy in 15 of the 18 datasets (83.33%) over other methods. The proposed approach also yielded the highest validation accuracy attainable in the COIL20 and Leukemia datasets which vividly portray the superiority of the BDMO.

## 1. Introduction

The data dimension significantly affects the Machine Learning (ML) model’s performance in data mining activities. In recent times, advanced devices that gather or generate data have made an enormous amount of data available in various application areas [1]. Although, in dealing with these huge and high-dimensional datasets, the major requirement is computational resources. Also, noise data like irrelevant and redundant features can significantly degrade the ML model’s performance. There is a need to remove these noisy features from the original dataset due to their ability to misinform the learning algorithm [2]. To this end, feature selection is imperative to settle the issue of dimensionality.

Feature selection (FS) is a search problem because it reduces the number of features from the original dataset without losing information [3]. The main aim of FS is to select feature subsets that best represent the dataset and show the most to the intended concepts. It does not just eliminate redundant or irrelevant data but also presents the benefit of interpretability and readability [4]. Feature selection can be grouped into filter and wrapper methods [5]. However, some researchers included a third category, embedded as found in [6]. The wrapper method predicts the accuracy of the already determined algorithm for learning to generate the selected features’ quality. It includes the classification algorithm, interacts with the classifiers, and yields a better result than the filter approach. The filter approach, on the contrary, isolates feature selection from the classifier learning and removes any bias of the learning algorithm from interfering with the feature selection’s algorithm (Aggarwal et al., 2014) [7]. It usually concentrates on the overall characteristics of the data [8] and does not involve a learning model in selection [9]. Examples of the filter method include t-test feature selection [10] and multivariate relative discrimination criterion [11]. The wrapper-based approach is the most preferred method for problems of classification.

Finding the optimal feature subsets in a wrapper-based technique is daunting because the goal is to choose the minimum number of subsets with the maximum accuracy. Based on the growing time required to locate the best feature subsets in a high-dimension dataset, feature selection is considered an NP-hard problem [12]. Should we have a dataset with *N* feature, we need a sum of 2^*N*^ features to investigate and locate the optima feature [13, 14]. Therefore, there is a need for a high-performing metaheuristic algorithm to reduce the processing time this kind of problem may pose.

The wrapper-based feature selection methods can be grouped into swarm intelligent, evolutionary-based algorithms, and physics-based algorithms. The inspiration for the swarm-based algorithms is often from the collective and foraging behavior of whales, ants, grasshoppers, fish, fireflies, and many other creatures in nature. Evolutionary-based approaches utilize the biological theory of evolution, such as mutation and crossover in nature. Physics-based methods mimic various laws of physics that generally occur in nature.

Dwarf Mongoose Optimisation (DMO) algorithm is a new swarm-based metaheuristic algorithm proposed by [15]. The DMO was developed on the principle of the social structure and foraging nature of dwarf mongooses in their natural environment. Since the algorithm was created, no variant of it has been proposed. The DMO algorithm was designed to solve continuous optimization problems in a continuous search space. Therefore, this binary version converts the search space into binary space and modifies the stepwise movement of the dwarf mongoose in the search space to solve the feature selection problem.

The major goal of the work is to harness the efficiency of the DMO algorithm to solve high-dimensional feature selection challenges. A binary variant of the DMO algorithm known as the Binary Dwarf Mongoose Optimisation (BDMO) is proposed to explore and find minimal feature subsets possible in high-dimensional datasets. The k-Nearest Neighbor (kNN) is used as the classifier to evaluate the selected feature subsets’ goodness. This proposed method was assessed using eighteen (18) high-dimensional datasets from the Arizona State University (ASU) feature selection repository. Additionally, ten well-known methods were utilized to ascertain the efficacy of the proposed BDMO. The main contributions of this work are summarized as follows:

- The introduction of binary approaches of the DMO algorithm called BDMO to select the smallest possible number of features from high-dimensional medical datasets.
- The binary DMO is achieved by adapting the main components of the standard DMO
- The binary search space was achieved by applying a low-cost and effective method where a threshold is assigned to each variable
- The proposed BDMO is evaluated and validated using eighteen (18) high-dimensional datasets from the Arizona State University (ASU) feature selection repository.
- The efficacy of the proposed FS method is compared with some other popular FS methods.

This article has seven sections. Section 2 presents a brief review of relevant literature, whereas the motivation for the study is presented in Section 3. The dwarf mongoose algorithm (DMO) is discussed in Section 4. Section 5 details the proposed BDMO approach and its application in feature selection. Section 6 centers on the results of the experiments and a discussion of the results. Finally, Section 7 concludes the work.

## 2. Related literature

Most metaheuristic algorithms are nature-inspired and can be categorized into four approaches based on the source of inspiration: The swarm-based algorithms are based on the cooperative and hunting behavior of whales, ants, grasshoppers, fish, fireflies, and a lot of other creatures in nature [16]. Swarm-based methods include artificial bee colony [17] and bat algorithms [18]. In the same vein, the evolutionary-based approaches utilize the biological theory of evolution, such as mutation and crossover. An example includes Corel reefs optimization [19].

On the other hand, physics-based methods mimic various laws of physics that generally occur in nature. Some physics-based examples include gravitational search algorithm and Equilibrium Optimizer [20, 21]. Different human activities inspire human-based methods, and teaching-learning-based optimization [22] is an example. These metaheuristic algorithms use exploitation and exploration activities to accomplish optimization.

The swarm-based methods are often biological systems that draw their inspiration from nature. The agents follow a simple procedure even though no central management structure controls how the individual agent is meant to behave [23]. Autonomy is a unique advantage of swarm-based algorithms because each agent represents a solution to a particular problem as they are not controlled by external management. Examples of algorithms in this category include Particle Swarm Optimization (PSO), Ant Colony Optimization, and Artificial Bee Colony (ABC) optimization.

Many proposed metaheuristic algorithms have provided optimal or near-optimal solutions to many real-world applications, including various feature selection problems [24]. Some of which include the Whale Optimization Algorithm (WOA) and its hybrid [16, 25, 26], Cuckoo Search Optimization Algorithm (CSO) [27–29], DragonFly Algorithm (DA) (Chantar et al., 2021; Cui et al., 2020; Sree Ranjini & Murugan, 2017) [30–32], Prairie Dog Optimization (PDO) Algorithm [33] and many more.

Particle swarm optimization (PSO) is the most prominent swarm-based algorithm. An improved binary version of the PSO was designed in 2008 by [34] to solve the gene selection problem. This approach resets the global best result if there is no improvement for three continuous iterations to cater to the premature convergence of the PSO. Two years later, [35] proposed a modified discrete PSO to solve the binary feature selection classification problem. The study used an adaptive selection of subsets in estimating the features’ relevant weight probability. The study [36] presented a catfish effect to solve binary feature selection challenges using the PSO. Should the global best get trapped in local optimal, a repositioned and reset was done on the weak nine-tenth particles.

In [37], a PSO-based feature selection was presented, which enhanced the efficacy of detecting skin cancer. Furthermore, Ji et al. [38] developed a co-evolution binary version of the PSO for feature selection. This method divided the population into sub-swarms and assigned various inertia weight strategies for diversity improvement. Banka & Dara [39] presented a hamming distance based on PSO to update the velocity for high-dimensional feature selection. The study showed the efficacy of this method in outperforming other traditional methods on three high-dimensional cancer datasets. The work by [40] presents another recent PSO-based approach to solving feature selection issues.

More swarm intelligence metaheuristic algorithms developed recently have their versions proposed to solve the feature selection problems. The grey wolf optimization algorithm (GWO) was inspired by the chasing procedure of a group of grey wolves in their natural environment [41]. The algorithm emulates the hierarchy of leadership and chasing approach of grey wolves in their natural setting. The GWO has been used recently for solving feature selection problems in data mining. Emary et al. [42] proposed a feature selection method based on multi-objective GWO in searching for the most appropriate and useful features. The hybrid approach employed the lower computation complexity in the filter method to advance the wrapper method’s performance. It was tested using different UCI datasets and achieved much robustness and stability.

Li et al. [43] proposed a novel predictive-based framework that hybridized an improved GWO (IGWO) and kernel extreme learning machine (KELM) known as IGWO-KELM and applied to problems in medical diagnosis. Moreover, Too et al. [44] proposed a novel viable binary variant of the grey wolf optimizer (CBGWO) to solve the feature selection challenge in the electromagnetic classification of signals. They extracted some time-frequency features from the STFT coefficient, and the new method was used to evaluate the optimal subset from the initial dataset. Sreedharan et al. [45] developed a system for recognizing facial emotion known as Facial Emotion Recognition (FER) that can analyze essential human facial expressions, like normal, smile, unhappy, angry, amaze, terrified, and irritate. The manner of recognition of the FER system was categorized into four activities, preprocessing, extraction of feature, selection of feature, and classification.

The authors in [46] presented a hybridization of a popular metaheuristic optimizer called GWO and a gradient descent algorithm which was used to resolve feature selection issues. Similarly, a newly proposed hybridized technique comprised the Extended Binary Cuckoo Search, Genetic Algorithm, and Whale Optimization Algorithm, which aimed to reduce the time required to search a huge database during image retrieval. This approach was compared with other popular classification algorithms like KNN, NB, Random Forest–RF, CatBoost, considering Recall, Precision, error rate, F-measure, etc. [47].

The Salp Swarm algorithm (SSA) developed by Mirjalili et al. [48] is another recently developed swarm-based metaheuristic algorithm. Two years after the SSA was developed, Ibrahim et al. [49] presented a hybridized optimization technique for feature selection problems. The proposed algorithm combined the SSA algorithm and the PSO called SSAPSO to improve the efficacy of both exploitation and exploration phases. A year later, Tubishat et al. [50] proposed a technique for selecting optimal feature subsets in the wrapper method and solving feature selection problems. They included two enhancements into the base SSA: Based Learning at the starting phase of SSA to improve its population diversity in the search space. Secondly, it included developing and using a new local search algorithm with SSA to enhance its exploitation.

In the same year, [51] developed a new version of SSA for feature selection known as the Improved Follower of Salp swarm Algorithm, which used the Sine Cosine algorithm and Disrupts Operator (ISSAFD), to update the followers’ position in the SSA by utilizing mathematical functions of sinusoidal as inspired from the Sine Cosine Algorithm (SCA). The enhancement improved the exploration phase and avoided getting stuck in the local zone. Hegazy et al. (2020) Hegazy et al. [52] improved the structure of basic SSA to enhance the solution accuracy, reliability, and convergence speed and was called ISSA. Inertia weight was added as a new control parameter to adjust the best solution. After that, Jain & Dharavath [53] presented a feature selection technique that improved the SSOA–Salp Swarm Optimization Algorithm called memetic–MSSOA, which they transformed into binary to get the best classification accuracy.

The evolution-based algorithms utilize the biological evolution theory like mutation and crossover in nature. The Genetic Algorithm (GA) developed by Holland [54] is a classic example in this category. The first time the GA was used in solving the feature selection problem was in 1993 [55]. Afterwards, Huang & Wang [56] employed the GA to solve the feature selection problem in synchronisation with the support vector machine (SVM) classifier. A few years later, Nemati et al. [57] proposed a hybridized GA with Ant Colony Optimisation (ANO) to select optimal subsets of features to predict protein function. After that, de Stefano et al. [58] utilized the GA for feature selection to solve handwriting recognition of characters. Rejer [59] designed an aggressive mutation and embedded it into the GA to solve the feature selection challenge in the brain-computer interface. In this approach, some sets of offspring were generated by each parent by mutating another gene of the chromosome that corresponds.

More recent works have also been conducted on feature selection as an optimization problem. [60, 61], the authors proposed a binary mantra ray foraging optimization and binary seagull optimizer to tackle the feature selection problem. Both studies adopted S and V-shaped transfer functions to binarize the baseline mantra ray foraging optimization and seagull optimization algorithms. The former created eight versions of the BMRFO, and the latter formed four versions of each method since the base algorithms were developed in continuous search space. The former study was evaluated using eighteen UCI repository datasets, and their results were compared with sixteen well-known methods. The authors reported that the proposed method outperformed other methods in the study regarding the number of features selected and classification accuracy, while the latter employed twenty-five benchmark functions to validate the performance of the BSOA. The study by [38] proposed an improved binary PSO (IBPSO) combined with levy flight as a local search technique to reduce the number of selected features and improve the classification accuracy. The study experimentation was conducted using sixteen classical datasets from the UCI repository. More so, Ma et al. [62] also created a binary hunger games search optimization algorithm (BHGSO) using the S and V-Shaped transfer function, which was evaluated on sixteen UCI datasets. The average classification accuracy of the result is 95% on most of the tested datasets. However, these related studies, except for the BHGSO, were not applied to high-dimensional datasets, which depict a real-world scenario to assess the robustness of the proposed methods.

As more nature-inspired methods emerge in the feature selection arena, Hichem et al. [63] presented a novel binary grasshopper optimization algorithm (NBGOA) to solve the feature selection optimization problem. The authors assessed their implementation using twenty-dimensional datasets and compared them with five popular feature selection problems. The study results showed a better performance in terms of the number of features selected, maximizing the accuracy of classification, and reduced computational time compared with five other state-of-the-art algorithms. Conversely, only three of the twenty datasets are high-dimensional. Meanwhile, our study employed all eighteen high-dimensional datasets with features varying from 1000 to over 22,000 from different categories. Remarkably, more state-of-the-art methods were compared with the BDMO, which portrays the efficacy of our proposed method in solving real-world problems.

## 3. Motivation

The past decades have witnessed how meta-heuristic algorithms have grown popular and proved their abilities in several optimization fields, including feature selection (FS) problems. The popularity can be attributed to the success of these algorithms in solving problems, which has also drawn lots of efforts in developing better-performing metaheuristic algorithms. FS-based optimization algorithms aim to find the optimal feature subset without information loss, an NP-hard problem. There is no actual solution to the FS problem. However, methods can be developed that find a better solution.

The No Free Lunch (NFL) theorem postulates that there is no guarantee that an algorithm would produce optimal results for other problems because it was able to find optimal results for some problems. The NFL means no one-size-fits-all algorithm exists for all optimization problems [64]. The reliance on this theory has driven research in this area. More researchers are coming up with high-performing metaheuristic algorithms for FS problems. The success of these FS-based metaheuristic algorithms motivated this study.

This study proposed a binary variant of the DMO called BDMO) is proposed to explore and find minimal feature subsets possible in high-dimensional datasets. The k-Nearest Neighbor (kNN) is used as the classifier to evaluate the selected feature subsets’ goodness. This classifier was selected due to its popular use in the FS domain and for its suitability in dealing with large dataset dimensions yielding higher classification accuracy than other classifiers [16, 65]. This proposed method was assessed using eighteen (18) high-dimensional datasets from the Arizona State University (ASU) feature selection repository. Additionally, ten well-known methods were utilized to ascertain the efficacy of the proposed BDMO.

## 4. Dwarf mongoose optimisation algorithm

The DMO is a member of the stochastic population-based metaheuristic algorithm developed by [15]. This algorithm mimicked the social and foraging behavior of the dwarf mongoose, also referred to as Helogale. The animals forage in groups, but individual dwarf mongoose does a thorough food search as feeding is not a collective exercise. Due to their seminomadic attribute, they build their sleeping mound close to an abundant food source and search for the next abundant food source. As shown in Eq (1), the DMO begins its update by initializing the mongoose’s candidate population. The population is stochastically generated between a particular problem’s lower bound (LB) and upper bound (UB).

$$X=[\begin{array}{lllll} x_{1,1} & x_{1,2} & \cdots & x_{1,d-1} & x_{1,d}\\ x_{2,1} & x_{2,2} & \cdots & x_{2,d-1} & x_{2,d}\\ \vdots & \vdots & x_{i,j} & \vdots & \vdots \\ x_{n,1} & x_{n,2} & \cdots & x_{n,d-1} & x_{n,d} \end{array}]$$ 1

where X is the set of the present population of candidates that are randomly generated using Eq (2), *x*_*i*,*j*_ indicates the position of the jth dimension of the ith population, n indicates the size of the population, and d refers to the problem dimension.

$$x_{i,j}=unifrnd(VarMin,\;VarMax,\;VarSize)$$ 2

where *unifrnd* is a random number that is uniformly distributed, *VarMin and VarMax* are lower bound and upper bound, respectively, *VarSize* is the problem dimension of the problem. So far, the best solution at every iteration is the best solution obtained.

**Fig 1:**
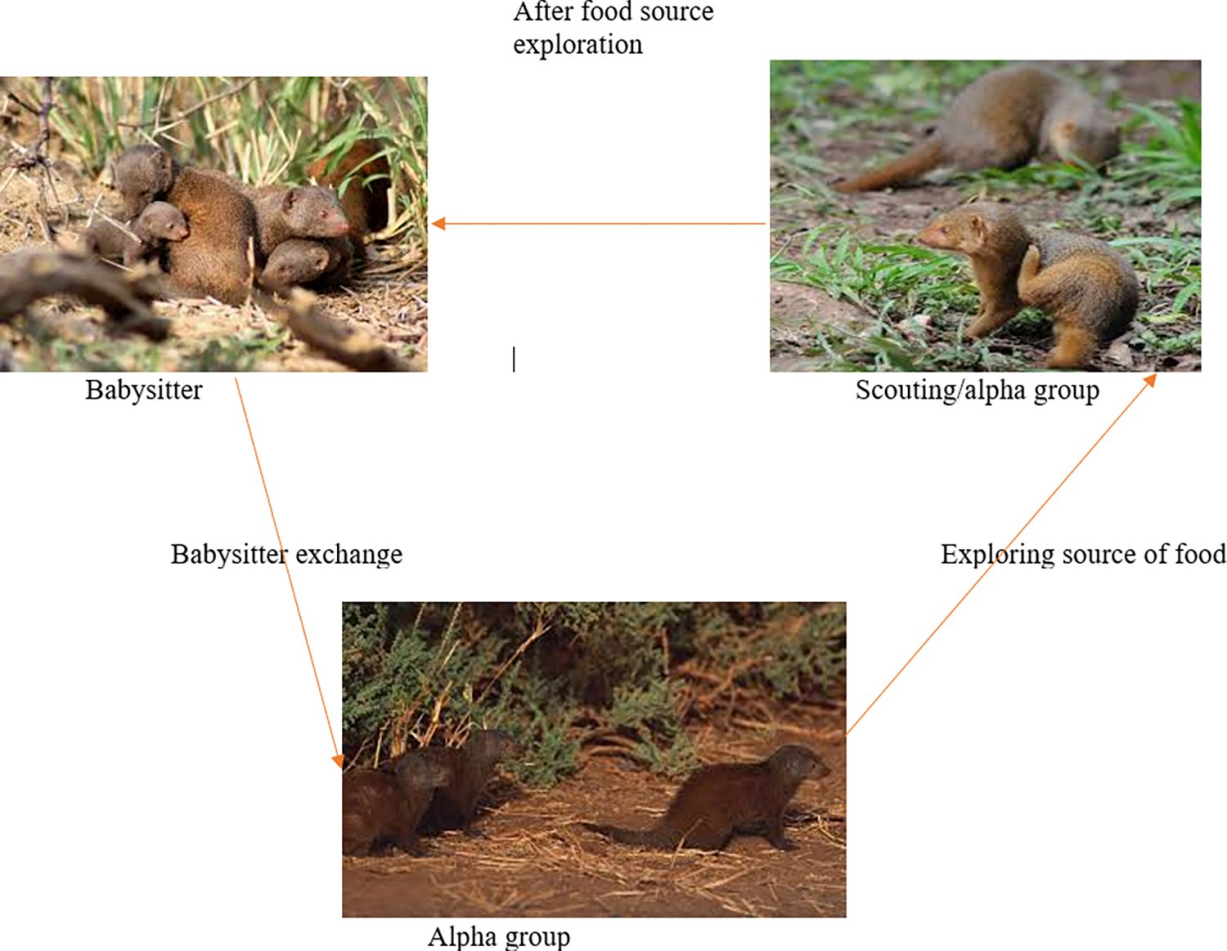
The optimization procedures of DMO [15].

Like the other metaheuristic algorithms, the DMO has two phases: exploitation (individual mongoose carry out a thorough search in a particular region) and exploration (a random search for a new abundant food source or new sleeping mound). The activities in the two phases are carried out by the three main social structures of the DMO: the alpha group, the scout group, and babysitters. The optimization step of the DMO algorithm is illustrated in Fig 1.

The alpha female (*α*) controls the rest of the family unit and is selected based on Eq 3.

$$\alpha =\frac{{fit}_{i}}{\sum_{i=1}^{n}{fit}_{i}}$$ 3

*n*−*bs* corresponds to the number of mongooses in the alpha group. Babysitters’ number is represented by *bs* while *peep* indicates the female alpha’s sound to ensure that the family is kept on the right path.

The abundant food source determines the sleeping mound position, and it is expressed in Eq 4 below.

$$X_{i+1}=X_{i}+phi*\mathrm{peep}$$ 4

where *phi* is a random uniformly distributed number [–1,1]. After every iteration, the sleeping mound is evaluated; Eq 5 represents the sleeping mound.


$${sm}_{i}=\frac{{fit}_{i+1}-{fit}_{i}}{max\{|{fit}_{i+1},{fit}_{i}|\}}$$ 5

An average value is given in Eq 6 when a sleeping mound is found.


$$\varphi =\frac{\sum_{i=1}^{n}{sm}_{i}}{n}$$ 6

As soon as the babysitter exchange criterium is attained, there is a movement to the scouting phase to evaluate the next sleeping mound, determined by the available food source.

The scout group searches for the next sleeping mound to ensure exploration since mongoose is known not to return to a previous sleeping mound. Foraging and scouting are done concurrently in DMOA with the rationale that the farther the family forage, the likelihood of locating the next sleeping mound simulated in Eq 7.

$$X_{i+1}=\left\{ \begin{array}{l} X_{i}-CF*phi*rand*{[X}_{i}-\vec{M}]\;if\;\varphi_{i+1}>\varphi_{i}\\ X_{i}+CF*phi*rand*{[X}_{i}-\vec{M}]\;else \end{array}\right.$$ 7

where *rand* is a random number between [0,1], $CF={\left(1-\frac{iter}{Max_{iter}}\right)}^{\left(2\frac{iter}{Max_{iter}}\right)}$ indicates the parameter that directs the collective-volatile movement of the mongoose’s group, which linearly reduces during iterations. $\vec{M}=\sum_{i=1}^{n}\frac{X_{i}\times {sm}_{i}}{X_{i}}$ denotes the vector which motivates the mongoose’s movement to another sleeping mound.

The babysitter’s group remains with the juveniles when the scouting and foraging group searches for a sleeping mound and food source. The number of members of this group is deducted from the total number of candidate population since they do not go foraging or scouting. However, when a certain parameter is met, as given in Eq 7, the babysitters exchange with the foraging or scouting group to search for food. Algorithm listing 1 presents the pseudocode for the standard DMO optimization algorithm,

**Algorithm 1 Pseudocode of the DMO**

***begin***

*Initialize the algorithm parameters*:

*[peep]*

*Initialize the mongoose populations (search agents)*: *n*

*Initialize the number of babysitters*: *bs*

*Set n = n-bs*

*Set babysitter exchange parameter L*

***For*** iter = 1: max_iter

    *Calculate the fitness of the mongoose*

    *Set time counter C*

    *Find the alpha based on Eq 3*

    $\alpha =\frac{{fit}_{i}}{\sum_{i=1}^{n}{fit}_{i}}$

    *produce a candidate food position using Eq 4*

    $X_{i+1}=X_{i}+phi*peep$

    *Evaluate new fitness of X*_*i*+1_

    *Evaluate sleeping mound using Eq 5*

    ${sm}_{i}=\frac{{fit}_{i+1}-{fit}_{i}}{max\{|{fit}_{i+1},{fit}_{i}|\}}$

    *Compute the average value of the sleeping mound found using Eq 6.*

    $\varphi =\frac{\sum_{i=1}^{n}{sm}_{i}}{n}$

    *Compute the movement vector using*

    $\vec{M}=\sum_{i=1}^{n}\frac{X_{i}\times {sm}_{i}}{X_{i}}$

    *Exchange babysitters if C*≥*L*, *and set*

    ${fit}_{i}=0$

    *Simulate the scout mongoose next position using Eq 7.*

    $X_{i+1}=\left\{ \begin{array}{l} X_{i}-CF*rand*{[X}_{i}-\vec{M}]\;if\;\varphi_{i+1}>\varphi_{i}\;Exploration\\ X_{i}+CF*rand*{[X}_{i}-\vec{M}]\;else\;Exploitation \end{array}\right.$

    *Update best solution so far*

***End For***

***Return*** *best solution*

***End***

## 5. The proposed approach

The DMO algorithm was utilized in solving engineering optimization problems. It outperformed other popular metaheuristic algorithms like Arithmetic Optimization Algorithm (AOA), PSO, Salp Swarm Algorithm (SSA), and Ant Colony Optimization (ACO) in solving some engineering problems. The efficacy of DMO in solving these global optimization issues motivated its binary version for solving feature selection challenges in this paper. In BDMO, the position of a dwarf mongoose can be seen as a feature subset. Every feature subset can have *N* features, where *N* happens to be the number of features in the original feature set. The fewer the number of selected feature subsets and the higher the accuracy of classification, the better the solution [66]. The proposed fitness function was used to evaluate each solution that relies on two main objectives: the number of feature subsets selected and the accuracy of the solution as produced by the classifier, KNN.

The algorithm commences with a population, the set of solutions generated randomly. The fitness function proposed is then used to assess each solution. The population’s fittest solution is represented as *BestSol* (Mongoose). DMO’s main loop is iterated a couple of times. In every iteration, the positions of the solutions are updated according to the foraging behaviors of the alpha group.

### 5.1. Binary dwarf mongoose optimization

In the dwarf mongoose optimization (DMO), the position vectors of the dwarf mongoose population are continuous values. In some peculiar issues, such as feature selection, solutions are restricted to binary values {0,1}. The approach was proposed to enhance the efficiency of the baseline DMO for high-dimensional feature selection issues. To tackle the feature selection problem, we represent the solution in binary form, 0 and 1. Usually, 1 represents the feature subset selected, while 0 denotes the unselected feature subsets. If, for instance, given solution *X* = {1,0,0,1,1,1,0,1,0,0}, this indicates selecting features in the first, fourth, fifth, sixth, and eighth position without selecting the others in the second, third, seventh, ninth, and tenth positions.

### 5.2. BDMO for feature selection

This section applies BDMO to high-dimensional datasets feature selection scenarios and classification issues. Feature selection is a necessary data preprocessing procedure to illustrate the best relevant, applicable, and essential feature space(s). This approach entails choosing a subset with the utmost discrete and appropriate feature(s) out of a huge class of features for record representation in a dataset for predictive modeling [67]. Practically, a traditional search that caters to all the feature spaces is unrealistic in application to high-dimensional datasets. Assuming there are 1000 features in total in a dataset, the probable number of solutions would be 2^1000^ = 1.071509*e*+301. Finding this number of subsets is daunting; therefore, the BDMO is used to solve this complex issue.

#### 5.2.1. Representation of the solution

For every solution, the dimension’s number *D* is the same as the features’ number, and therefore, each dimension in the dataset indicates the index of the corresponding feature. For example, if a solution has 2000 dimensions, 2000 features are contained in the solution.

In every solution, the limit of the dimension is in the range of [0, 1]. The static threshold of 0.5 is utilized to ascertain if a feature is to be selected or not, as shown in Eq 8 below. For a feature to be selected, the position index must be 0.5 and above, which rounds the value to 1, and any feature with the position index of less than 0.5 is rounded down to 0 and will not be selected.

$${BestSol}^{d}=\left\{ \begin{array}{c} {BestSol}_{i}^{d}>0.5\;feature\;is\;selected\\ {BestSol}_{i}^{d}\leq 0.5\;unselectedfeature \end{array}\right.$$ 8

where ${BestSol}_{i}^{d}$ is the best solution *i* in dimension *d*. Thereby, a mongoose’s position shows that a feature set is selected as the value of position increases for the dimensions [42].

### 5.3. Fitness function

To simplify this study, we employ the classification error rate (CEE) as the fitness function in assessing the performance of selected features using the solution. The calculation of fitness function (Fit) is given below:

$$↓Fit=CEE=\frac{Number\;of\;wrongly\;classified}{Total\;number\;of\;instances}$$ 9

The CEE denotes the classification error rate in the kNN (*kNN*, *k* = 5) algorithm (Emary & Zawbaa, 2019; Xue et al., 2014) [42, 68]. In kNN, the Euclidean distance (ED) used to measure k neighbor’s distance is defined by [69] as:

$$ED(Y,X)=\sqrt{\sum_{d=1}^{D}}{\left(X^{d}-Y^{d}\right)}^{2}$$ 10

where *X* and *Y* indicate the specific features in an instance and *D* signifies the total number of features used. The best reduct of the wrapper-based technique was generated using the kNN classifier *where K* = 5 [70]. In cross-validation for assessment, every dataset in this proposed method is divided into training and testing samples of 80% and 20%, respectively. The training samples were utilized for feature selection evaluation, while the remaining hidden samples were employed to test [71]. This paper utilized straight cross-validation with *K* = 10 to resolve the over-fitting challenges. This validation method partitioned the training samples into tenfold equal size first. After this, the 9 (*k*−1) were used as training set for the classifier, and the last one-fold utilised for validation information. The process of evaluation was repeated ten times which replaces the training and validation folds. The different results of the average data rounds are recorded. The updating equation of the alpha group is a function of the movement vector $\vec{M}$ that is calculated in Eq 8. The pseudocode for the proposed BDMO is shown in algorithm listing 2.

**Algorithm 2 Pseudocode of the BDMO**

**Input:** Searchagent_no, Max_iter, fs

***begin***

*Initialize the algorithm parameters*:

*[peep]*

*Initialize BDMO population from feature set* ***fs***

*Let* ***AlphaGroup*** *= fs[*:***Searchagent_no****]*

***bs = fs[Searchagent_no*:*]***

***nAlphaGroup = size(AlphaGroup)***

***no_bs = size(bs)***

*Set babysitter exchange parameter L*

*Evaluate the fitness of each* ***fs***, ***AlphaGroup***

*Define the best solution*, ***BestSol*** *from* ***fs***

***while*** *maximum iteration (****Max_iter****) is not reached*

    *Select a set of dimension rates using tournament selection*

    *Calculate the fitness of the mongoose*

    $↓Fit=CEE=\frac{Number\;of\;wrongly\;classified}{Total\;number\;of\;instances}$

    *Set time counter C*

    *produce a candidate food position using Eq 4*

    ${Bestfs}_{i+1}={Bestfs}_{i}+phi*peep$

    *fit*_*i*_ = *Evaluate new fitness of Bestfs*_*i*+1_ *using Eq 8*

    ${Bestfs}^{d}=\left\{ \begin{array}{c} {Bestfs}_{i+1}>0.5\;feature\;is\;selected\\ {Bestfs}_{i+1}\leq 0.5\;unselectedfeature \end{array}\right.$

    *Evaluate sleeping mound using Eq 5*

    ${sm}_{i}=\frac{{fit}_{i+1}-{fit}_{i}}{max\{|{fit}_{i+1},{fit}_{i}|\}}$

    *Compute the average value of the sleeping mound found using Eq 6.*

    $\varphi =\frac{\sum_{i=1}^{n}{sm}_{i}}{n}$

    *Compute the movement vector using*

    $\vec{M}=\sum_{i=1}^{n}\frac{{fs}_{i}\times {sm}_{i}}{{fs}_{i}}$

    *Exchange babysitters if C*≥*L*, *and set*

    ${fit}_{i}=0$

    *Simulate the scout mongoose next position using Eq 7.*

    ${Bestfs}_{i+1}=\left\{ \begin{array}{l} {Bestfs}_{i}-CF*rand*{[Bestfs}_{i}-\vec{M}]\;if\;\varphi_{i+1}>\varphi_{i}\;Exploration\\ {Bestfs}_{i}+CF*rand*{[Bestfs}_{i}-\vec{M}]\;else\;Exploitation \end{array}\right.$

    *Update best solution* ***Bestfs***

    *Store Bestfs*^*d*^

  ***end for***

***end while***

***Output*** *Global best solution*, ***Bestfs*,** *all* Bestfs^d^

**Fig 2:**
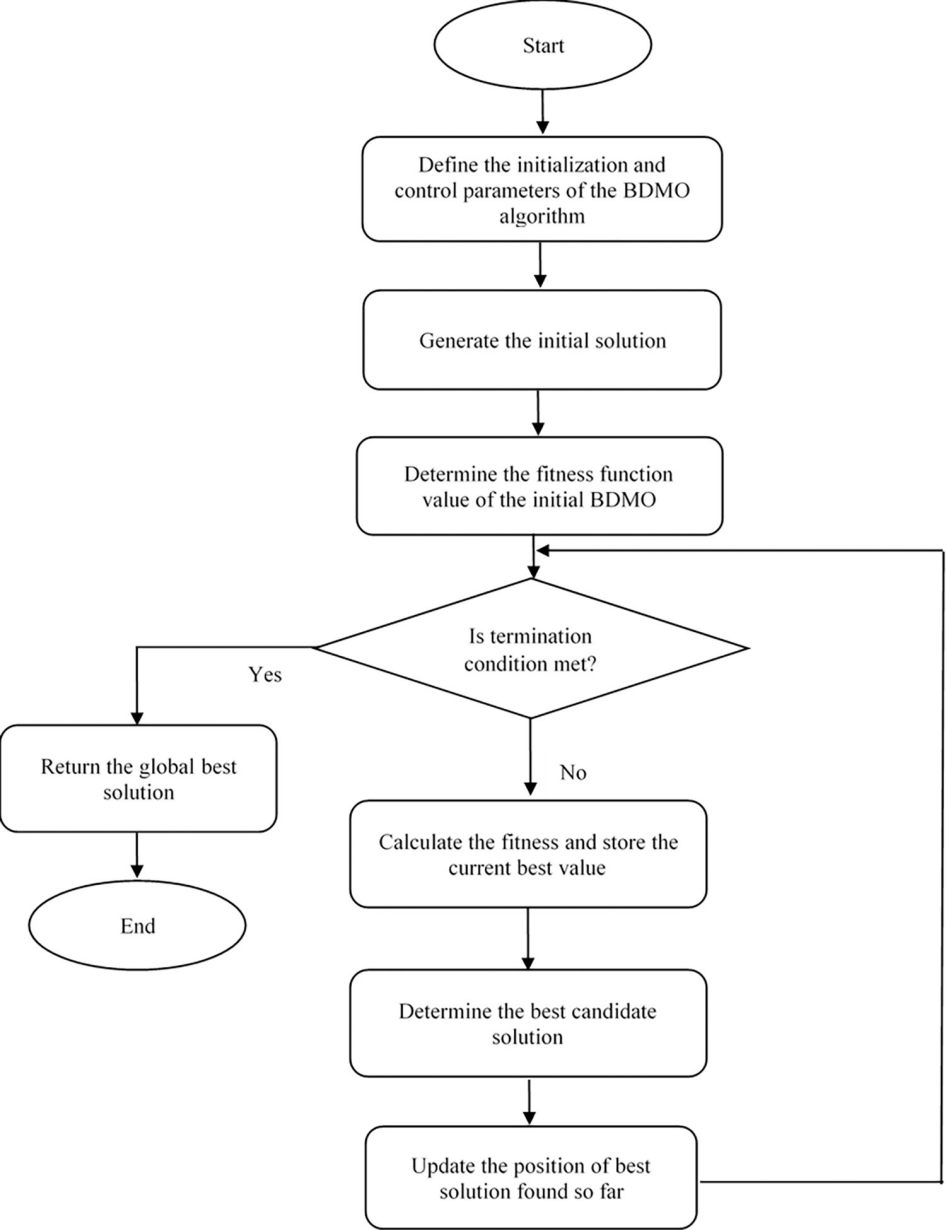
Flowchart depicting the structure of the BDMO algorithm.

The steps of optimization of the proposed BDMO algorithm to solve the FS problem are shown in Fig 2. This figure begins its step with parameter definition followed by generating its initial population representing the feature selection problem’s set of solutions. After that, each candidate solution’s fitness function depends on evaluating and selecting the best features. Then, the identification and retention of the current best solution are made. Next, the BDMO algorithm updates the current population using either Eq 7 or 8, which also depends on the fitness function’s quality. The process is designed so that if the fitness function’s probability of the current solution is higher than 0.5, Eq 7 is chosen for the update. Contrarywise this, and the current solution is updated by Eq 8. Notably, the probability stated is the position index’s computation factor (Position index) > = 0.5. Subsequently, each solution’s fitness function is the computation of Eq 9, and after the population is updated, the best solution is established. The BDMO then checks that the stopping criteria are met. If so, the algorithm returns the overall best solution candidate. Conversely, the algorithm then repeatedly performs the previous steps by checking whether the Position index is > = 0.5 until it reaches the final stop condition.

## 6. Experimental results and discussion

This section presents the experimental setup and discusses the results and discussions.

### 6.1. Dataset (High-dimensional)

Eighteen high-dimensional datasets were obtained from the Arizona State University feature selection repository to evaluate this proposed method’s performance. The details of the employed datasets, including their feature number, classes, instances, and categories, are presented in Table 1. Each dataset comprises many features of not less than one thousand (1000) and is multiclass, ranging from 2 to 20 classes. High-dimensional datasets often represent real-world situations and are also more challenging. Not most metaheuristic algorithms perform satisfactorily with high-dimensional and multiclass data.

**Table 1:** Dataset and their properties.

Number	Datasets	# features	# instances	# Classes	Categories
**1**	ALLAML	7129	72	2	Biological
**2**	CLL-SUB-111	11,340	111	3	Biological
**3**	COIL20	1024	1440	20	Face image
**4**	Colon	2000	62	2	Biological
**5**	GLA-BRA-180	49,151	180	4	Biological
**6**	GLI-85	22,283	85	2	Biological
**7**	GLIOMA	4434	50	4	Biological
**8**	Leukemia	7070	72	2	Biological
**9**	Lung	3312	203	5	Biological
**10**	Lymphoma	4026	96	9	Biological
**11**	Nci9	9712	60	9	Biological
**12**	Orlraw10P	10,306	100	10	Face image
**13**	Prostate_GE	5966	102	2	Biological
**14**	SMK-CAN-187	19,993	187	2	Biological
**15**	TOX-171	1748	171	4	Biological
**16**	warpAR10P	2400	130	10	Face image
**17**	warpPIE10P	2420	210	10	Face image
**18**	Yale	1024	165	15	Face image

### 6.2. Experimental setup

The proposed binary DMO algorithm was implemented using MATLAB. To assess the efficacy of the proposed technique, ten well-known approaches: Spatial bound whale optimization algorithm (SBWOA), Simplified spatial bound whale optimization algorithms (S-SBWOA) [44], Binary Particle Swarm Optimization (BPSO), Jaya algorithm (JA), crow search algorithm (CSA), cooperative swarm optimizer (CSA), moth flame optimization (MFO), hamming distance-based binary particle swarm optimization (HDBPSO), salp swarm algorithm (SSA), and generalized normal distribution optimization (GNDO) were compared with the BDMO.

The experiment of this study ran twenty (20) times and evaluated each method two hundred times for every dataset. The choice of 20 independent runs of the respective algorithms is premised on the belief that it will give enough room to measure the stability of the algorithms. After rigorous parametric analysis, the parameters for the proposed method are set as follows in all experiments: the population size is ten (10) and one hundred (100) iterations. The proposed method performed better with a small population size and the number of iterations, hence our choice of the set parameters. The selected optimizers’ population size and the number of iterations are also the same for fair comparison [30, 66]. All algorithms implement the same fitness function. The computer specification for this implementation is Core i7, 3.60GHz CPU with 16GB RAM. Other parameter settings presented in Table 2 are as reported by their respective authors.

**Table 2:** Experiment’s parameter setting.

Parameter	Value
K-fold cross-validation number	10
Agent number	10
Number of runs	20
Maximum iterations	100
Dimension of problems	Features’ number in the dataset
Length of CSA flight	1.5
CSO’s social factor	0.2
CSA’s awareness probability	1.5
MFO’s Parameter **b**	1
HDBPSO’s acceleration factors	2
Parameters **c1, c2** in BPSO	2,2

### 6.3. Results and analysis

This sub-section presents the results produced by this proposed approach. The criteria below were used to assess the proposed method:

- The standard deviation and mean of the fitness values obtained from various methods are presented.
- The proposed and competitive techniques’ validation and testing accuracies are also presented.
- The average number of features selected from each dataset across the 20 runs is presented.
- The convergence curve of the proposed method is presented.
- The average time of computation of all runs is shown.
- The Wilcoxon sign-rank test of BDMO and other techniques are stated.

#### 6.3.1. Comparison of the proposed method with other state-of-the-art methods

In this sub-section, the goal is to compare the performance of the proposed method with other well-known methods such as the Spatial bound whale optimization algorithm, SBWOA & S-SBWOA [44], BPSO [72], JA [22], CSO [73], CSA [74], MFO [75], HDBPSO [39], SSA [76], and GNDO [77].

Tables 3 and 4 report the fitness values’ mean and standard deviation for BDMO and other algorithms used for the comparison. A critical look at the results presented in Table 3 shows that the BDMO is efficacious at finding the exact minima. These best fitness values are bolded in the tables. Compared with the rival methods, the BDMO produced the optimal mean fitness for most datasets (14 datasets of 18). The performance of the BDMO can be attributed to the effective search mechanism adapted from DMO and the low-cost and effective method used to convert the continuous search space of DMO to binary search space [78]. The BPSO was next competitive as it produced optimal mean fitness values in 6 datasets, SBWOA in 3, and S-SBWOA in 1 dataset. Friedman’s test was used to rank the significance of the algorithms based on their performance in minimizing fitness, as is shown in Table 3. The BDMO ranked first, closely followed by SBWOA.

**Table 3:** Mean fitness values.

No	Datasets	BDMO	SBWOA	S-SBWOS	JA	MFO	BPSO	CSA	CSO	GNDO	SSA	HDBPSO
**1**	ALLAML	**0.0714**	0.0827	0.0970	0.1387	0.1317	**0.0714**	0.1453	0.1443	0.1385	0.1532	0.1673
**2**	CLL-SUB-111	**0.1818**	0.2609	0.2719	0.3240	0.3138	**0.1818**	0.3476	0.3408	0.3320	0.3612	0.3926
**3**	COIL20	**0.0003**	0.0045	NA	NA	NA	0.0010	NA	NA	NA	NA	NA
**4**	Colon	**0.0833**	0.0980	0.1003	0.1538	0.1395	NA	0.1665	0.1585	0.1548	0.1693	0.1988
**5**	GLA-BRA-180	**0.1931**	**0.1917**	NA	NA	NA	0.1944	NA	NA	NA	NA	NA
**6**	GLI-85	**0.0588**	0.0717	0.0775	0.0967	0.0967	**0.0588**	0.1092	0.1092	0.1050	0.1183	0.1442
**7**	GLIOMA	0.20	**0.1275**	0.1358	0.1688	0.1658	0.20	0.1721	0.1767	0.1688	0.1817	0.1979
**8**	Leukemia	0.0321	0.0337	0.0397	0.0725	0.0693	**0.0107**	0.0840	0.0812	0.0772	0.0858	0.1003
**9**	Lung	0.0388	**0.0207**	0.0217	0.0280	0.0260	0.045	0.0295	0.0287	0.0301	0.0320	0.0394
**10**	Lymphoma	0.1184	0.0651	**0.0624**	0.0798	0.0755	0.1263	0.0799	0.0829	0.0799	0.0820	0.0910
**11**	Nci9	**0.1667**	0.4695	0.4793	0.5487	0.5348	**0.1667**	0.5570	0.5590	0.5482	0.5690	0.5973
**12**	Orlraw10P	**0.050**	0.060	0.0621	0.1036	0.1021	**0.050**	0.1043	0.1057	0.1036	0.1057	0.1136
**13**	Prostate_GE	**0.05**	0.0945	0.1053	0.1278	0.1242	0.145	0.1363	0.1363	0.1339	0.1425	0.1602
**14**	SMK-CAN-187	**0.0554**	0.2372	0.2468	0.2713	0.2629	0.0622	0.2828	0.2740	0.2770	0.2870	0.3033
**15**	TOX-171	**0.1177**	0.2221	0.2317	0.2346	0.2275	0.1368	0.2704	0.2454	0.2454	0.2717	0.3142
**16**	warpPIE10P	**0.0476**	0.1129	0.1167	0.1371	0.1354	0.1179	0.1476	0.1423	0.1385	0.1521	0.1665
**17**	warpAR10P	**0.2442**	0.3878	0.4121	0.4837	0.4774	0.2789	0.5055	0.4965	0.4946	0.5092	0.5426
**18**	Yale	**0.2681**	0.3499	0.3632	0.3823	0.3641	0.2833	0.3960	0.3767	0.3869	0.3998	0.4297
Friedman’s test mean rank		2.53	3	3.47	5.77	4.5	4.2	8.1	7.67	6.67	9.43	10.67
Rank		1	2	3	6	5	4	9	8	7	10	11

**Table 4:** Standard deviation of fitness values.

No	Datasets	BDMO	SBWOA	S-SBWOS	JA	MFO	BPSO	CSA	CSO	GNDO	SSA	HDBPSO
**1**	ALLAML	**0**	0.0380	0.0290	0.0364	0.0343	**0**	0.0361	0.0346	0.0393	0.0346	0.0326
**2**	CLL-SUB-111	**5.6953e-17**	0.0336	0.0346	0.0367	0.0342	**5.6953e-17**	0.0335	0.0343	0.0399	0.0382	0.0344
**3**	COIL20	**0.0016**	0.0032	NA	NA	NA	**0.0016**	NA	NA	NA	NA	NA
**4**	Colon	**4.2715e-17**	0.0369	0.0324	0.0425	0.0434	NA	0.0466	0.0515	0.0442	0.0474	0.0544
**5**	GLA-BRA-180	0.0062	0.0188	NA	NA	NA	**8.543e-17**	NA	NA	NA	NA	NA
**6**	GLI-85	**0**	0.0484	0.0277	0.0361	0.0361	**0**	0.0380	0.0447	0.0416	0.0452	0.0469
**7**	GLIOMA	**8.543e-17**	0.0311	0.0600	0.0628	0.0577	**8.543e-17**	0.0578	0.0607	0.0621	0.0669	0.0788
**8**	Leukemia	0.0365	**0.0231**	0.0287	0.0371	0.0361	0.0262	0.0339	0.0403	0.0379	0.0349	0.0385
**9**	Lung	0.0128	0.0101	0.0088	0.0091	**0.0083**	0.0103	0.0084	0.0093	0.0098		
**10**	Lymphoma	0.0234	0.0153	**0.0151**	0.0220	0.0231	0.0265	0.0232	0.0248	0.0213	0.0236	0.0251
**11**	Nci9	**5.6953e-17**	0.0520	0.0606	0.0670	0.0586	**5.6953e-17**	0.0599	0.0624	0.0623	0.0599	0.0578
**12**	Orlraw10P	**0**	0.0183	0.0193	0.0207	0.0224	**0**	0.0218	0.0238	0.0240	0.0224	0.0243
**13**	Prostate_GE	**0**	0.0197	0.0346	0.0254	0.0260	0.0154	0.0271	0.0261	0.0259	0.0220	0.0256
**14**	SMK-CAN-187	**0.0060**	0.0265	0.0327	0.0423	0.0384	0.0127	0.0404	0.0434	0.0425	0.0405	0.0410
**15**	TOX-171	**0.0234**	0.0385	0.0360	0.0389	0.0330	0.0239	0.0358	0.0270	0.0271	0.0347	0.0299
**16**	warpAR10P	**0.0188**	0.0474	0.0331	0.0583	0.0617	0.0246	0.0613	0.0607	0.0647	0.0312	0.0311
**17**	warpPIE10P	**0**	0.0243	0.0243	0.0284	0.0267	0.0053	0.0298	0.0375	0.0268	0.0617	0.0637
**18**	Yale	0.0178	0.0331	0.0291	0.0445	0.0413	**0.0148**	0.0385	0.0375	0.0347	0.0355	0.0438

The bolded values in Table 3 depict the best mean fitness values obtained in the experiment. For instance, in datasets 1 to 6 and 11 to 18, the BDMO produced the least mean fitness values, showing its efficacy over other methods in the experiment. The next competitive method is the BPSO with the same values as the BDMO on 4 occasions, beating the BDMO in 1 instance. The SBWOA produced a better fitness value mean on 2 datasets and S-SBWOA on 1 dataset. The bolded values in Table 4 show the best standard deviation values obtained in the experiment. For example, in datasets 1 to 4, 6 & 7, and 11 to 17, the BDMO produced the least standard deviation, showing its efficacy over other methods in the experiment. The next competitive method is the BPSO which ties with the BDMO on 6 occasions and beats the BDMO on 2 datasets. The SBWOA and S-SBWOA could produce better standard deviation on 1 dataset each.

BDMO shows a high consistency and strength compared to other methods by generating the smallest standard deviation value in 14 cases out of 18, which portrays remarkable performance in resolving high-dimensional feature selection issues. For example, on ALLAML, GLI-85, Orlraws10P, Prostate_GE, and warPIE10P datasets, the proposed BDMO produced 0, which is the smallest value obtainable as against BPSO, which is its closest rival with the same value on ALLAML, GLI-85, and Orlraws10P. Finally, the BDMO is the best performing high-dimensional feature selection algorithm to locate the global optimum, leading to suitable performance.

**Fig 3:**
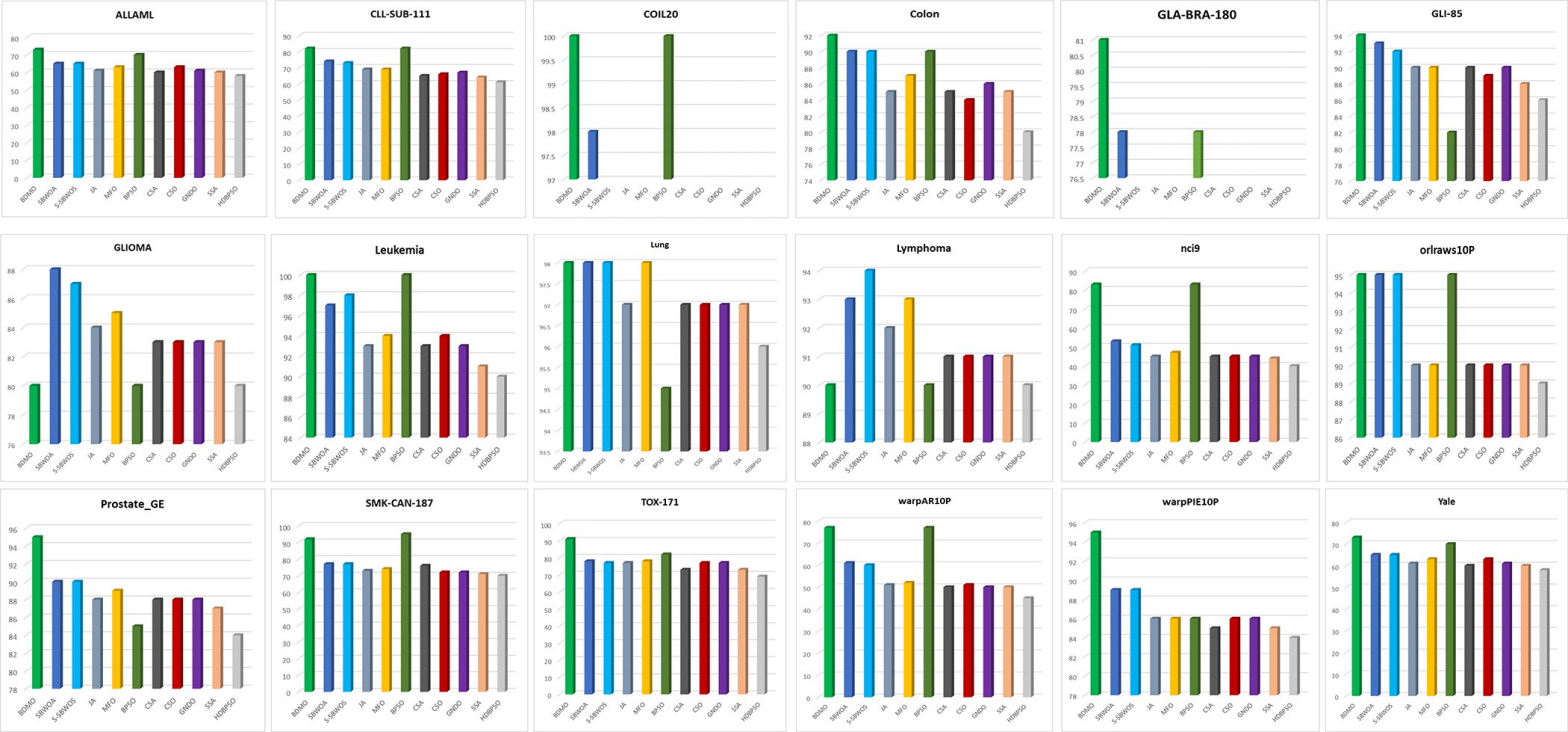
Comparison between the proposed BDMO and the state-of-the-art methods based on accuracy validation on all selected high-dimensional feature selection datasets.

In Fig 3, the results of validation accuracy are illustrated. The BDMO outperforms other methods in producing exceptional values of validation accuracy in 15 of 18 datasets. Moreover, the BPSO is next, producing exceptional values in 7 datasets, S-SBWOA and SBWOA were the best in 2 datasets. Finally, MFO recorded a tie with three other methods on the Lung dataset. In 2 datasets (COIL20 and Leukemia), the proposed BDMO generated the highest achievable validation accuracy of 100%, while the BPSO produced the same accuracy rate in the Leukemia dataset. Based on test results, the proposed BDMO performed competitively, which implies that our proposed method could explore the untried feature space to locate the optimum feature sets, enabling it to generate the highest accuracies on most occasions.

**Fig 4:**
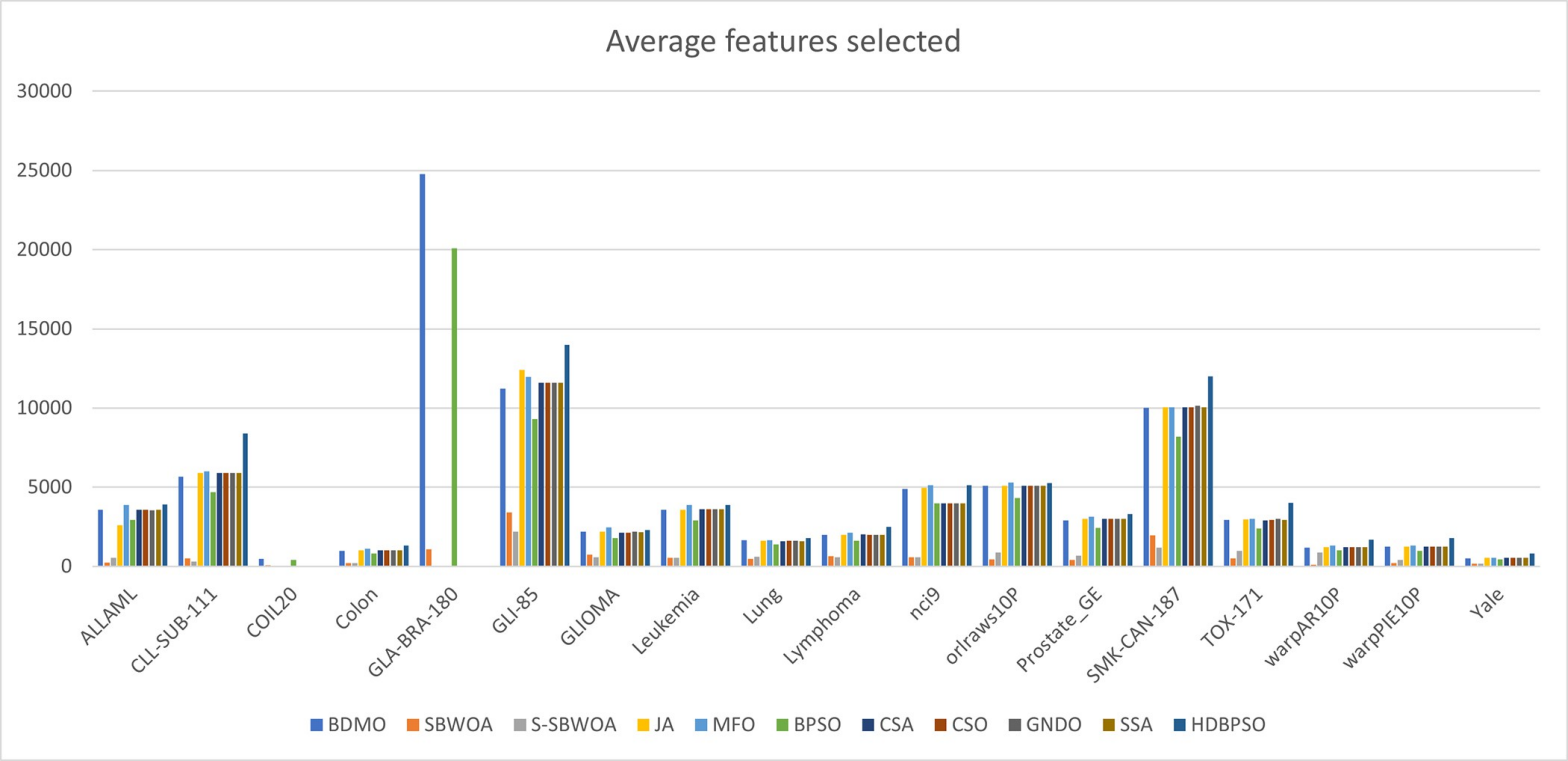
Average feature selected by BDMO and other approaches.

Fig 4 displays the average feature subsets selected. The results show that the S-SBWOA and SBWOA selected significantly lower features than the BDMO and other methods. Since the BDMO produced the highest prediction accuracy in 83% of the case, we can therefore infer that there may be information loss with these methods. For instance, on the GLA-BRA-180 dataset, SBWOA selected approximately 1,100 features subsets from over 49,000 features, whereas BDMO selected 24,788 features. On GLI-85 with over 22,000 features, S-SBWOA and SBWOA selected approximately 2,200 and 3,400, respectively, and the BDMO selected 11,220, which supports our assumption of information loss. In another case, the BPSO, the main competitor, produced less feature size than our proposed method. However, the BDMO selected fewer features in many cases than the other seven methods. For this reason, we intend to improve the ability of the proposed BDMO to reduce its computational cost in future research.

#### 6.3.2. Convergence analysis

**Fig 5:**
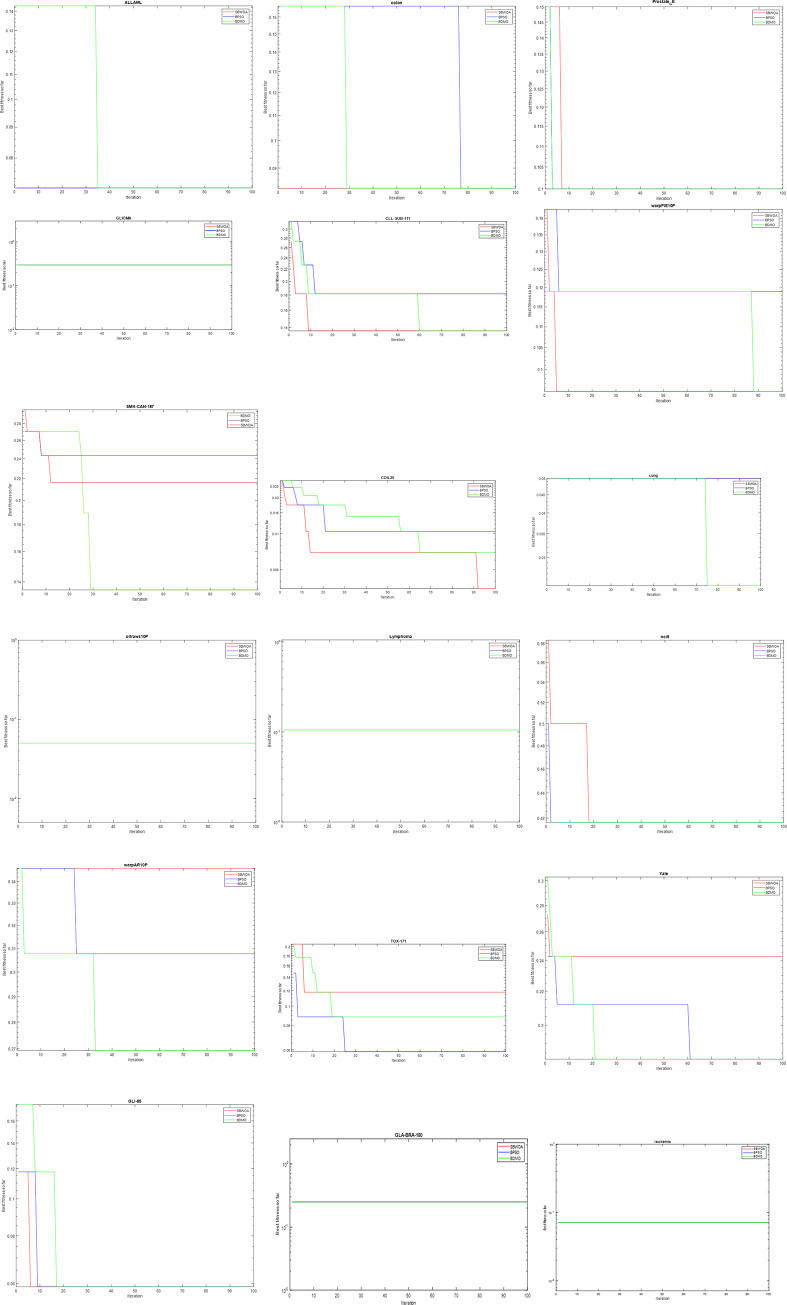
Illustration of the convergence curves for the three most prominent approaches employed in this study, namely BDMO, BPSO, and SBWOA to solve all the selected high-dimensional feature selection datasets.

The analysis of the convergence behavior of the BDMO, BPSO, and SBWOA, which are the best performing methods in this study, was reported in this subsection. This analysis focuses on how the three methods behave when employed to solve the high-dimensional optimization problem of feature selection. Fig 5 depicts the outcome of convergence curves for the three most prominent approaches in this study. The figure indicates that DBMO converged faster and deeper. This is because it found the optimum solution early in the iteration process. Also, the robustness and stability of BDMO ensure that it stays near or at the optimal solution as the optimization progresses. The figure also shows that the BDMO improves the solution throughout the iteration process. Among these three approaches, the SBWOA’s convergence rate was not as good as the proposed method and BPSO.

#### 6.3.3. Computational time

Another area of consideration in feature selection is computation speed, particularly in higher dimensional situations. The average computation cost for the proposed approach with other competitive methods is shown in Table 5. It can be vividly noticed that the SBWOA and S-SBWOA have a higher computation speed than the BDMO. The BDMO competed with other methods in finding optimal feature sets in considerably less time, although not as fast as the SBWOA and S-SBWOA, which has the mechanism to compress the population size and can reduce the solution number in later iterations. The added computational cost of the BDMO arises from the DMO’s process of alpha selection and the number of objective function evaluations. This is a limitation to be improved on in the future. Even though the proposed approach performed excellently to get higher validation accuracy, least average, and standard deviation of the fitness values, it consumes more computational time than the two methods in this paper in a high-dimensional scenario.

**Table 5:** Average time of computation of BDMO and other approaches (in seconds).

No	Datasets	BDMO	SBWOA	S-SBWOS	JA	MFO	BPSO	CSA	CSO	GNDO	SSA	HDBPSO
1	ALLAML	11.2	3.1	**2.2**	14.4	14.5	13.9	13.4	8.8	28.0	13.2	24.6
2	CLL-SUB-111	18.8	5.8	**4.2**	32.0	31.7	22.1	29.1	18.9	56.3	28.8	54.5
3	COIL20	74.7	**23.7**	NA	NA	NA	52.8	NA	NA	NA	NA	NA
4	Colon	6.1	1.4	**0.9**	2.4	2.5	5.8	2.4	1.8	4.7	2.3	3.9
5	GLA-BRA-180	108.8	**76.4**	NA	NA	NA	119.3	NA	NA	NA	NA	NA
6	GLI-85	25.5	14.8	**8.7**	48.2	47.6	35.9	46.6	31.4	92.8	45.0	88.2
7	GLIOMA	6.8	2.1	**1.4**	3.6	3.8	8.2	3.4	3.0	7.0	3.3	10.5
8	Leukemia	10.5	3.2	**2.2**	13.9	14.5	12.8	13.8	8.7	27.3	13.4	24.8
9	Lung	14.1	4.5	**3.1**	18.5	18.6	12.6	18.0	10.2	32.1	16.4	30.0
10	Lymphoma	9.8	2.7	**1.8**	5.5	9.4	10.3	5.4	3.8	11.0	5.0	19.0
11	Nci9	10.8	3.6	**2.5**	15.6	16.4	15.1	15.0	10.9	29.3	14.9	27.9
12	Orlraw10P	15.9	5.3	**3.5**	27.0	27.2	19.1	24.6	15.7	50.1	23.6	48.4
13	Prostate_GE	12.1	3.4	**2.5**	16.8	17.0	13.6	15.7	10.2	32.0	15.3	27.2
14	SMK-CAN-187	53.5	22.6	**14.3**	94.9	95.2	53.3	89.7	53.1	183.1	88.4	174.6
15	TOX-171	17.6	6.2	**4.6**	26.7	25.3	17.1	24.5	13.5	48.7	24.2	41.7
16	warpAR10P	8.1	2.1	**1.5**	4.5	4.6	0.7	4.5	2.9	7.8	4.0	14.7
17	warpPIE10P	12.2	3.5	**2.3**	14.1	14.0	10.5	13.9	7.2	26.4	13.3	22.9
18	Yale	6.9	2.1	**1.4**	3.2	3.2	5.8	3.1	1.9	6.2	3.0	4.4

#### 6.3.4. Validation accuracy and F-measure

Fig 4 shows the validation accuracy of our proposed method and other methods in the study. In 15 out of 18 cases, the BDMO produced the highest validation accuracies over other methods. Our proposed approach also generated the highest accuracy values of 100% on two (Colon and Leukemia) datasets. The BPSO is usually the biggest rival with 7 best validation values and produced 100% accuracy on the 1 (Leukemia) dataset. S-SBWOA is next in validation accuracy results on 2 datasets, SBWOA and MFO on 1 dataset. The bolded values in Table 6 show the best precision values obtained in the experiment. For example, the BPSO produced the highest precision values in 15 out of 18 datasets. This is followed by the BDMO, which yielded the highest precision values, 12 out of 18, and CSA on 1 dataset. To further test the results of experiments in this study, the F-measure test was conducted with the values in Table 7 above. The BPSO also outperformed in this test by producing 15 highest results out of 18 datasets employed in this study. The BDMO followed closely by yielding 12 highest F-measure values out of 18 datasets and the S-SWOA on one occasion. These consistent results show the potency of our proposed approach in solving the problem of feature selection in high-dimensional cases.

**Table 6:** Precision of BDMO and other approaches.

No	Datasets	BDMO	SBWOA	S-SBWOS	JA	MFO	BPSO	CSA	CSO	GNDO	SSA	HDBPSO
**1**	ALLAML	**1**	0.8442	0.8328	0.8142	0.8211	**1**	0.8121	0.8099	0.8082	0.8126	0.8115
**2**	CLL-SUB-111	**1**	0.6955	0.6802	0.6652	0.6483	0.7	0.6410	0.6440	0.6625	0.6546	0.6552
**3**	COIL20	**0.9890**		NA	NA	NA	**0.9890**	NA	NA	NA	NA	NA
**4**	Colon	0.875	0.8162	0.8202	0.8072	0.7953	**1**	0.7930	0.8050	0.7987	0.8132	0.8062
**5**	GLA-BRA-180	0.8214	0.6667	NA	NA	NA	**0.8276**	NA	NA	NA	NA	NA
**6**	GLI-85	**0.9091**	0.7026	0.6896	0.6812	0.7031	**0.9091**	0.6699	0.6924	0.7063	0.7175	0.6975
**7**	GLIOMA	0.625	0.7644	0.7562	0.7702	0.7681	0.625	**0.7725**	0.7652	0.7663	0.7479	0.7681
**8**	Leukemia	**0.9**	0.8873	0.8993	0.8851	0.8917	**0.9**	0.8856	0.8916	0.8892	0.8977	0.8882
**9**	Lung	**1**	0.9426	0.9358	0.9486	0.9508	**1**	0.9491	0.9207	0.8832	0.9489	0.9333
**10**	Lymphoma	**0.875**	0.9366	0.9319	0.9429	0.9371	**0.875**	0.9383	0.6278	0.6395	0.9333	0.6344
**11**	Nci9	**0.9**	0.6721	0.6404	0.6518	0.6405	0.8	0.6463	0.2964	0.2876	0.6658	0.2805
**12**	Orlraw10P	**0.9444**	0.9362	0.9411	0.9412	0.9403	**0.9444**	0.9420	0.9255	0.9275	0.9390	0.9255
**13**	Prostate_GE	**0.9091**	0.8772	0.8641	0.8661	0.8742	**0.9091**	0.8727	0.8674	0.8618	0.8729	0.8691
**14**	SMK-CAN-187	**0.7391**	0.6572	0.6751	0.6466	0.6532	**0.7391**	0.6533	0.6585	0.6571	0.6608	0.6489
**15**	TOX-171	0.9091	0.7035	0.6731	0.6900	0.6705	**1**	0.6570	0.6750	0.6697	0.6672	0.6640
**16**	warpAR10P	0.6364	0.5986	0.5789	0.5900	0.5998	**0.8235**	0.5916	0.5233	0.5039	0.5905	0.5091
**17**	warpPIE10P	**0.8919**	0.8807	0.8902	0.8675	0.8638	**0.8919**	0.8813	0.8642	0.8719	0.8725	0.8681
**18**	Yale	0.8214	0.7020	0.6779	0.7060	0.7017	**0.8462**	0.7015	0.6251	0. .6217	0.6976	0.6447

**Table 7:** F-measure of BDMO and other approaches.

No	Datasets	BDMO	SBWOA	S-SBWOS	JA	MFO	BPSO	CSA	CSO	GNDO	SSA	HDBPSO
**1**	ALLAML	0.75	0.8915	**0.8972**	0.8799	0.8885	0.75	0.8822	0.8780	0.8784	0.8819	0.8818
**2**	CLL-SUB-111	**0.8235**	0.6505	0.6430	0.6041	0.5852	**0.8235**	0.5823	0.5814	0.5992	0.5923	0.5943
**3**	COIL20	**0.9945**		NA	NA	NA	**0.9945**	NA	NA	NA	NA	NA
**4**	Colon	0.875	0.8322	0.8334	0.8225	0.8091	**0.9333**	0.8159	0.8129	0.8111	0.8274	0.8231
**5**	GLA-BRA-180	0.8364	0.7692	NA	NA	NA	**0.8421**	NA	NA	NA	NA	NA
**6**	GLI-85	**0.9091**	0.7316	0.7047	0.7203	0.7454	**0.9091**	0.7194	0.7259	0.7493	0.7714	0.7326
**7**	GLIOMA	**0.7692**	0.7225	0.7128	0.7240	0.7312	**0.7692**	0.7283	0.7312	0.7269	0.7182	0.7348
**8**	Leukemia	**0.9474**	0.9129	0.9314	0.9163	0.9269	**0.9474**	0.9220	0.9231	0.9235	0.9264	0.9198
**9**	Lung	0.9167	0.8447	0.8599	0.9009	0.8741	**0.96**	0.8826	0.8917	0.8635	0.8807	0.9066
**10**	Lymphoma	**0.8235**	0.6157	0.6308	0.6576	0.6373	**0.8235**	0.6540	0.6436	0.6555	0.6242	0.6488
**11**	Nci9	**0.9**	0.3334	0.2645	0.3049	0.2993	0.8421	0.2980	0.3037	0.2998	0.2933	0.2899
**12**	Orlraw10P	**0.9714**	0.8939	0.8998	0.8997	0.8974	**0.9714**	0.8970	0.8903	0.8935	0.8939	0.8903
**13**	Prostate_GE	**0.9524**	0.8665	0.8319	0.8331	0.8437	**0.9524**	0.8489	0.8356	0.8312	0.8422	0.8406
**14**	SMK-CAN-187	**0.8095**	0.6179	0.6159	0.6013	0.6073	**0.8095**	0.6070	0.6141	0.6049	0.6052	0.6084
**15**	TOX-171	0.8511	0.6711	0.6428	0.6556	0.6389	**0.8889**	0.6265	0.6393	0.6354	0.6386	0.6302
**16**	warpAR10P	0.7180	0.4690	0.4578	0.4343	0.4198	**0.9714**	0.4273	0.4317	0.4290	0.4232	0.4260
**17**	warpPIE10P	**0.9429**	0.8585	0.8705	0.8442	0.8435	**0.9429**	0.8597	0.8407	0.8506	0.8484	0.8442
**18**	Yale	**0.8519**	0.5428	0.5289	0.5449	0.5447	0.8302	0.5369	0.5236	0.5267	0.5362	0.5356

### 6.4. Wilcoxon rank test

The experimental results obtained are tested statistically using Wilcoxon’s test and presented in Table 8. From the results, the BDMO significantly outperforms the SBWOA, S-SBWOA, JA, MFO, BPSO, CSA, CSO, GNDO, SSA, and HDPSO on most of the datasets judging by the positive ranks returned by the BDMO. Also, the BPSO was competitive, judging by the number of ties returned between its comparison. At a significance level set at α = 0.05, Wilcoxon’s test showed a significant difference in all cases, which implies that the BDMO significantly outperformed all the algorithms.

**Table 8:** Results of Wilcoxon sign test on validation accuracy.

Algorithms		N	Mean Rank	Sum of Ranks	Z	Asymp. Sig. (2-tailed)
SBWOA—BDMO	Negative Ranks	15	8.80	132.00	-2.627b	0.009
	Positive Ranks	2	10.50	21.00		
	Ties	1				
	Total	18				
S-SBWOS–BDMO	Negative Ranks	13	8.08	105.00	-2.556b	0.011
	Positive Ranks	2	7.50	15.00		
	Ties	1				
	Total	16				
JA—BDMO	Negative Ranks	14	9.36	131.00	-3.258b	0.001
	Positive Ranks	2	2.50	5.00		
	Ties	0				
	Total	16				
MFO—BDMO	Negative Ranks	13	8.73	113.50	-3.039b	0.002
	Positive Ranks	2	3.25	6.50		
	Ties	1				
	Total	16				
BSPO—BDMO	Negative Ranks	9	5.67	51.00	-2.395b	0.017
	Positive Ranks	1	4.00	4.00		
	Ties	8				
	Total	18				
CSA—BDMO	Negative Ranks	14	9.36	131.00	-3.258b	0.001
	Positive Ranks	2	2.50	5.00		
	Ties	0				
	Total	16				
CSO—BDMO	Negative Ranks	14	9.36	131.00	-3.258b	0.001
	Positive Ranks	2	2.50	5.00		
	Ties	0				
	Total	16				
GNDO—BDMO	Negative Ranks	14	9.36	131.00	-3.258b	0.001
	Positive Ranks	2	2.50	5.00		
	Ties	0				
	Total	16				
SSA—BDMO	Negative Ranks	14	9.36	131.00	-3.258b	0.001
	Positive Ranks	2	2.50	5.00		
	Ties	0				
	Total	16				
HDBPSO–BDMO	Negative Ranks	14	8.50	119.00	-3.351b	<,001
	Positive Ranks	1	1.00	1.00		
	Ties	1				
	Total	16				

b. Based on positive ranks.

Categorically, the BDMO outperformed or was competitive in 90% of all cases. The results also confirmed the searchability, stability, and efficiency of the BDMO in solving the feature selection optimization problems used in this study. The performance of BDMO was not hindered by the characteristics associated with the feature selection problems, which is choosing the optimal number subset of features that will guarantee high performance. This performance can be attributed to the balanced exploitation and exploration introduced by each optimization phase of the DMO.

### 6.5. Discussion

It can be stated clearly from the results gotten that the proposed BDMO outperforms other methods in terms of accuracy and its ability to find the best subset of features, which shows its superiority over some well-known methods like S-SBWOA, SBWOA, JA, BPSO, MFO, SSA, CSO, CSA, GNDO, and HDBPSO. Although the BDMO selected a good number of features, it did not perform excellently in terms of computational time compared to SBWOA and S-SBWOA. This method also showed its strength in producing the smallest mean, standard deviations of the fitness values, and confirmed by the precision & F-measure values obtained with the p-value of the Wilcoxon test conducted, shown in Tables 4 to 7.

Overall, the conclusion can be drawn that the BDMO significantly increased efficiency in handling the task of high-dimensional feature selection. The performance of BDMO can be attributed to the optimization process of the DMO, where the fittest mongoose is selected as the alpha in a generation. The remaining mongooses gravitate toward the alpha in the next generation, and a new alpha is selected continuously until the end of the optimization process. The search space is effectively covered by the choice of movement steps of DMO to avoid being trapped in local optima. Furthermore, the ability of mongooses to scout for an abundant source of food and sleeping mound without returning to the previous sleeping mound increases the probability of selecting good dimension boundaries. By taking advantage of this mechanism, the BDMO selected features that can considerably boost classification accuracy. In general, we can infer that the BDMO is a potent tool for higher dimension feature selection and can be employed in application areas that have to do with higher dimensional data, like the field of medicine, where medical records increase regularly.

With the power of this proposed approach comes its limitations. The first observable shortcoming is the higher computational time of the BDMO compared to 2 of the competitive (the S-SBWOA and SBWOA) approaches in this study. We utilized the kNN classifier as a learning algorithm to validate performance. However, in future work, we intend to employ other popular classifiers like Support Vector Machine (SVM), Decision Tree (DT), Random Forest (RF), and Neural Networks (NN), which may come with an additional cost of computation. Also, the strategy of population compression can be employed to improve the cost of computation of the BDMO.

## 7. Conclusion

This study proposed a binary variant of the newly developed Dwarf Mongoose optimization algorithm called BDMO to handle high-dimensional feature selection challenges. This proposed method leverages the advantages and properties of the DMO in employing local and global search behaviors. Eighteen (18) high-dimensional datasets were employed to validate this approach. Then the proposed approach was compared with other popular methods. The results showed that our proposed method is reliable and efficient in handling high-dimensional optimization problems in feature selection. The proposed method has also overtaken its competitors, considering its fitness values. The proposed method also produced the highest accuracy, closely followed by the BPSO, SBWOA, and S-SBWOA. The BPSO produced the highest values for F-measure and precision for the largest percentage of datasets, although our BDMO closely followed it. The precision and F-measure were utilized to confirm the results produced by our method with a competitive result with its closest rival, the BPSO. Eventually, our proposed approach will be a suitable tool in the clinical and medical fields where high-dimensional data are generated frequently, and higher data are involved in the diagnosis.

The BDMO, as presented, only converted the continuous search space of the DMO to suit the binary search space in feature selection problems. However, the optimization process of the BDMO can be improved to solve the problem of the high number of features selection encountered in the course of this study. Future efforts can be made to modify or hybridize the BDMO with other well-known state-of-the-art population-based optimization algorithms. Also, some form of intelligence or machine learning capabilities can be incorporated into the BDMO to improve its performance for solving complex real-world application problems in different domains.
